# A person-centered integrated care quality framework, based on a qualitative study of patients’ evaluation of care in light of chronic care ideals

**DOI:** 10.1186/s12913-018-3246-z

**Published:** 2018-06-20

**Authors:** Gro Berntsen, Audhild Høyem, Idar Lettrem, Cornelia Ruland, Markus Rumpsfeld, Deede Gammon

**Affiliations:** 1 Norwegian center for eHealth research, University Hospital of Northern Norway, PB. 35, 9038 Tromsø, Norway; 2 Department of primary care, Institute of community medicine, UIT – The Arctic University of Norway, PB 6050, Langnes, 9037 Tromsø, Norway; 3 Center for Shared Decision Making and Collaborative Care Research, Oslo University Hospital, Sogn Arena, Pb 4950 Nydalen, N-0424 Oslo, Norway; 4 Department of Integrated Care, University Hospital of Northern Norway, PB. 35, 9038 Tromsø, Norway; 5 General Practice Health Centre, 9050, Storsteinnes, Norway; 6 Department for Internal Medicine, University Hospital of Northern Norway, PB 101, 9038 Tromsø, Norway

**Keywords:** Person-centered care, Delivery of healthcare, Integrated care, Health service research, Multimorbidity, Long-term conditions, Evaluation research, Care process, Goal attainment, Continuity of care

## Abstract

**Background:**

Person-Centered Integrated Care (PC-IC) is believed to improve outcomes and experience for persons with multiple long-term and complex conditions. No broad consensus exists regarding how to capture the patient-experienced quality of PC-IC. Most PC-IC evaluation tools focus on care events or care in general. Building on others’ and our previous work, we outlined a 4-stage goal-oriented PC-IC process ideal: 1) Personalized goal setting 2) Care planning aligned with goals 3) Care delivery according to plan, and 4) Evaluation of goal attainment. We aimed to explore, apply, refine and operationalize this quality of care framework.

**Methods:**

This paper is a qualitative evaluative review of the individual Patient Pathways (iPP) experiences of 19 strategically chosen persons with multimorbidity in light of ideals for chronic care. The iPP includes all care events, addressing the persons collected health issues, organized by time. We constructed iPPs based on the electronic health record (from general practice, nursing services, and hospital) with patient follow-up interviews. The application of the framework and its refinement were parallel processes. Both were based on analysis of salient themes in the empirical material in light of the PC-IC process ideal and progressively more informed applications of themes and questions.

**Results:**

The informants consistently reviewed care quality by how care supported/ threatened their long-term goals. Personal goals were either implicit or identified by “What matters to you?” Informants expected care to address their long-term goals and placed responsibility for care quality and delivery at the system level. The PC-IC process framework exposed system failure in identifying long-term goals, provision of shared long-term multimorbidity care plans, monitoring of care delivery and goal evaluation. The PC-IC framework includes descriptions of ideal care, key questions and literature references for each stage of the PC-IC process. This first version of a PC-IC process framework needs further validation in other settings.

**Conclusion:**

Gaps in care that are invisible with event-based quality of care frameworks become apparent when evaluated by a long-term goal-driven PC-IC process framework. The framework appears meaningful to persons with multimorbidity.

**Electronic supplementary material:**

The online version of this article (10.1186/s12913-018-3246-z) contains supplementary material, which is available to authorized users.

## Background

For persons with multiple long-term conditions and complex healthcare needs, diagnosis centered, fragmented and reactive care is believed to cause a poorer care experience and worse outcomes [1–3]. Our current healthcare system owes much of it’s success to a reductionist and specialized approach where we understand each diagnosis by its cause and treatment. As a result, a person with multimorbidity will receive care from a multitude of specialists who either cater to a part of the body (e.g., neurology) or provide one type of treatment (e.g., surgery). However, patients experience care within the context of their life and not through the professional lens of a diagnosis or treatment modality. For instance, persons with multimorbidity report how care involving multiple providers induces the experience of being an incidental carrier of many diagnoses or being a messenger between diagnosis specific professionals. They report confusion amidst numerous single disease treatments, which are rarely reviewed together [4–6]. In the context of multimorbidity, it is therefore reasonable and necessary to study the entire set of healthcare activities generated by all care providers within the larger frame of the person and his/her life project.

### Person-centered and integrated care

Multimorbidity guidelines increasingly identify both Person-Centered and Integrated Care (PC-IC) as central components of quality of care [7–10]. A PC-IC goal-oriented approach also has strong traditions in fields that commonly work with patients over the long-term, such as rehabilitation, geriatrics and General Practice [11, 12]. A PC-IC process is believed to enhance both technical and patient-experienced quality of care to produce the triple aim [13] of improved care experience, health, and function, as well as cost-benefit ratios [14–21]. Despite the widespread agreement of the desirability of PC-IC, progress in this area seems to be slow [5, 22–25]. The lack of progress may be due to the unclear conceptualization of what PC-IC is, and absence of evaluation tools that support improvement efforts. The literature on PC-IC is awash with overlapping and conflicting concepts and terminology [26–29], making it challenging to develop united frameworks that may structure patients’ experiences of care quality. PC-IC represents quality dimensions that are best assessed by the patient. Both person-centered care and integrated care concepts pertain to how the multi-faceted care system creates a seamless, personalized pathway that addresses the person’s needs, values and preferences as they develop over time.

To come to grips with this complexity, we have simplified the overall concept of patient-experienced quality of care using PC-IC as a starting point. We were inspired by; the practice of goal-oriented chronic care [11, 12], our knowledge of theoretical concepts and models relevant to chronic care [30–33], principles for goal-directed process design [34], and experiences from our previous research on goal setting [35]. The result is a 4-stage goal-oriented PC-IC cyclical process.

### The PC-IC cyclical process

1. Goal identification: Our starting point is a goal-oriented definition of PC-IC, where the person’s overarching goals drive decisions about care [31, 35]. The rationale for the goal-oriented approach is simple: A person perspective requires a strong element of care coordination to ensure that all contributors work towards a common goal. It is only the person, both in ethical and legal terms, who can legitimately identify what the overarching goal should be. It is not enough to be respectful and attentive, nor is it enough to involve and engage the person. PCC is a matter of transferring power to whatever the person has identified as his/her overarching goal. Together with the person, the professionals make this the real driver for decision making [35]. Some patients may not wish or be able to participate in such a process. Care professionals may then have to seek advice from the person’s significant others or make intelligent guesses about “what matters” to the person. The point is to make the overarching goal(s) for care explicit. If they remain unspoken, participants lose the “coordinating effect” of a common goal. Also, mismatches between patient preferences and health care assumptions may be missed by both patients and professionals.
2. Care planning: The personalized goals are used to identify the multidisciplinary team needed to assess the patient’s health issues. Subsequently, the team produces a comprehensive care plan aimed at goal attainment [7–10]. The plan should as far as possible be evidence-based [9] and should support health literacy [36], patient involvement and self-management [14]. The plan should identify roles, tasks, and responsibilities, including those taken on by the person and his/her significant others, to ensure seamless care.
3. Care delivery: The team, including the person, is then responsible for care delivery according to the plan. Loyalty to the plan is essential, as a plan that is not carried out, or a derailed plan will not produce the desired outcomes. Included in care delivery is the regular review of goals, plan and goal attainment whenever needed.
4. Goal evaluation: Patient-driven evaluation of goal-attainment, is the last of the four stages of the cycle. Goal evaluation serves as feedback to all contributors in the seamless care process. The result should be documented and linked back to goal adjustment and learning for the next cycle, in line with complexity and quality improvement theory [12, 37, 38].

We have called the timeline map of all care events for one individual the “individualized Patient Pathway” (iPP). The iPP borrows the “pathway” term and the timeline from the “care pathways” concept, which maps out events designed to manage a single condition across providers [39]. A multimorbidity iPP will thus represent the aggregate of all care delivery meant to care for the person’s ensemble of health issues, organized by time. The ideal iPP follows the rules similar to project management: Participants share clear goals. Plans detail sub-goals and sub-tasks. The resources needed to achieve goals are identified and allocated. Goal attainment indicates success. Finally, the project adjusts goals before the cycle starts over.

### Knowledge gaps and aim for the study

Many authors have already tried to make quantitative instruments that evaluate the patient care experiences in light of chronic care ideals [1, 40–45]. However, the underlying assumption in these instruments is that the care event is the basic unit of interest. We have not found evaluation frameworks that make the long-term iPP the unit of study. When the process, as opposed to the event, comes into focus, the goal of the process becomes the success criterion. Previous qualitative studies exploring iPP experiences exist [5, 46–51], but none of these compare care to an ideal. To the best of our knowledge, reviewing the iPP experience through the “lens” of the above PC-IC framework is unexplored.

Norway, like most other western nations, is pursuing large-scale transformation towards PC-IC, which has been an explicit health policy since the late 1990s [52, 53]. This focus has become even more prominent with the current administration [54]. The Norwegian healthcare system has comparable medical outcomes to other western healthcare systems [55]. A systematic exploration of a new method for PC-IC evaluation of the iPP in a Norwegian context should therefore be of general interest.

Our primary aim was to explore how the PC-IC process ideal might be useful as a guide to capture iPP quality and then apply, refine and operationalize this ideal into a quality of care framework. Our research questions were:

- What can we learn about the patient-experienced quality of care by the application of a PC-IC ideal to 19 iPP experiences of persons with multimorbidity?
- Which lessons from the empirical analysis can contribute to the refinement and operationalization of the PC-IC ideal into an evaluation framework?

## Methods

**Fig. 1:**
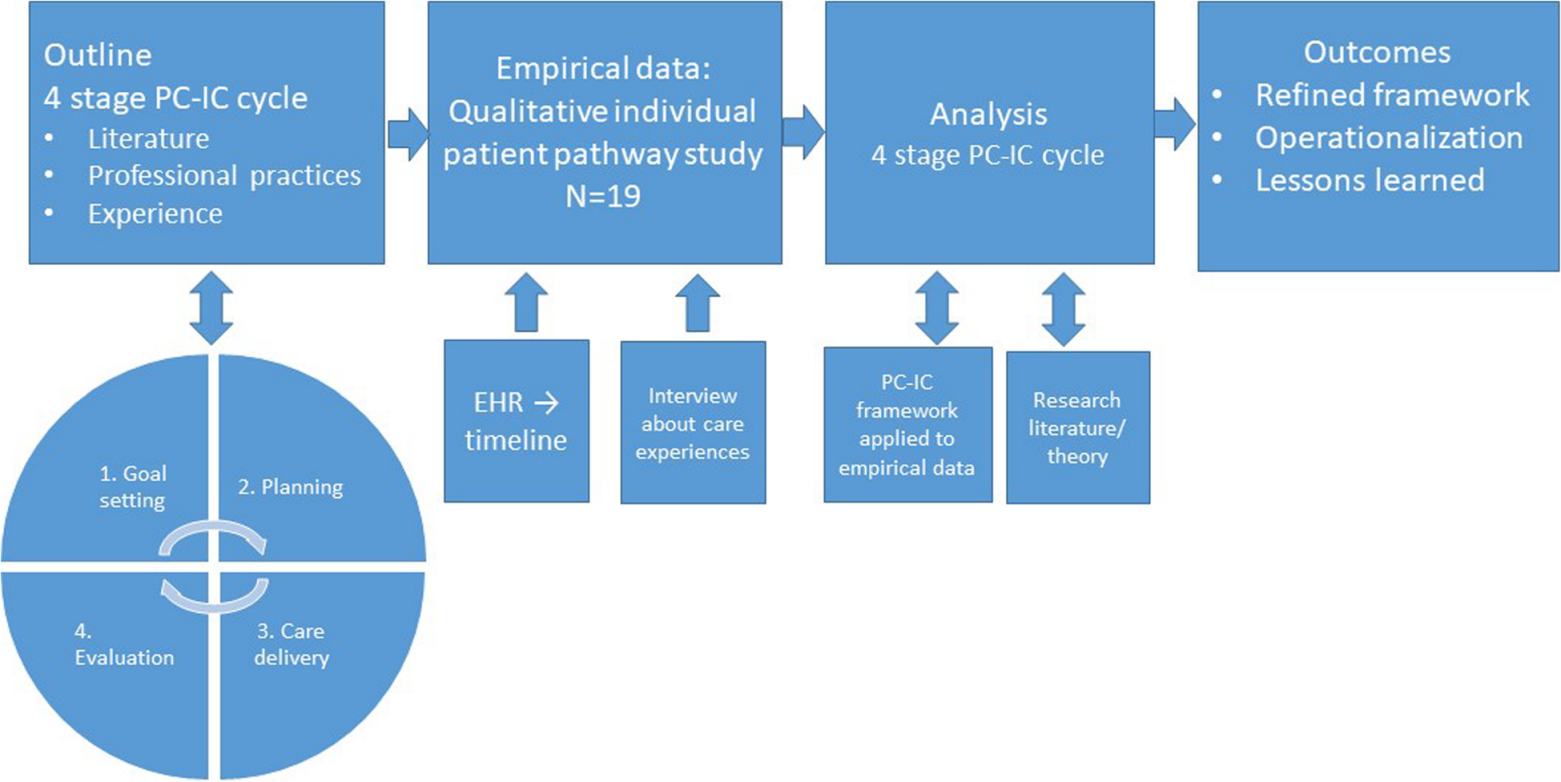
An overview of the stages of the research process included in this paper

Our work is a qualitative evaluative study of patient-experienced quality of care relative to PC-IC ideals for chronic care, conducted within a pragmatic interactionist tradition [56–58]. While we acknowledge that central concepts of disease, health, and care are socially constructed, we treat these constructs as stable and familiar enough to illuminate how patients evaluate their care [56]. We applied the PC-IC ideal as a structuring framework to the iPP-experience of 19 strategically selected individuals ad modum Ritchie [59]. An outline of the research process is given in Fig. 1.

### Material

#### Setting, informants, and recruitment

We aimed to include informants with a wide range of experiences with long-term health challenges. Cancer patients in active treatment represented experiences of a severe long-term life-threatening condition with a clear starting point and treatment options. Cancer survivors with ongoing sequelae represent patients with considerable everyday health challenges, but fewer treatment options. Finally, persons with various long-term conditions and complex needs served to examine similarities across diagnoses. We refer to study participants as either persons, informants or participants. The term “patient” in this text, refers to the smaller part of a person’s life when he/she is in direct interaction with a healthcare provider.

The material incorporates data from 19 persons with long-term, complex care needs from two studies:

Study 1: Thirteen persons (age 48–74) with cancer under active treatment, or cancer survivors with long-term sequelae, taking part in the Connect study (online cancer support) [60], were recruited by the local cancer nurse. One participant died, and one withdrew leaving 11 persons in the project.

Study 2: In the Troms-Ofoten (TO) study, care providers and patient advocacy organizations purposively selected one person from each of the following eight groups to ensure diversity regarding condition(s), context and demography. 1) Frail elderly with an episode of emergency care 2) Diabetes 3) Cardiovascular/pulmonary disease 4) Mental health issues 5) Cancer 6) Child with multiple disabilities 7) Mental health and substance abuse 8) Postoperative care. Age 9–76 years. Methods and preliminary results for the TO-study can be found in a Norwegian language report [61] (Table 1).

**Table 1:** Background characteristics and care complexity measures for 19 informants, Norway, 2011–2013

Informant background	N	N	N
Gender	8 males	11 female	
Employment status	7 employed	7 unemployed	1 child/4 pensioners
Living arrangements	3 alone	16 with spouse/children	
Home municipality	14 rural	5 urban	
Care complexity	Mean	Median	Range
# of diagnoses treated per year	5	4	(2–10)
# different health services per year	6	5	(2–12)
# of general practice visits per year	10	7	(1–36)
# of health service visits per year	28	21	(5–132)
# of inpatient days per year	16	4	(0–130)

#### Data collection

All informants filled a questionnaire on their socioeconomic and demographic background.

##### *Timeline of iPP*

We constructed the iPP timeline of clinical events from the hospital, general practice (GP) and nursing services^1^ electronic health record (EHR) from the prior 6^1^ or 12^2^ months. We created a table timeline of all clinical events, defined as consultations for diagnostic, therapeutic, or informational purposes. For each event we recorded time, EHR source (hospital, nursing service or GP), type of contact (outpatient, phone, admission), place (geographical, organizational), health profession, the degree of urgency and a short text summary of the main issue of the event. We produced simple quantitative descriptions (means, median, and range) of pathway complexity normalized for follow up time.

##### *The patient interview*

We used the iPP timeline as a basis for the retrospective, evaluative interview with each informant. The interviews followed a semi-structured interview guide (see online Additional file 1) which briefly included:

1. A shared review of the iPP timeline. We invited the informant to:
  - Correct any mistakes. (No corrections made).
  - Identify events of importance, along with their reasons for identifying them as such. Follow up questions elicited the informants’ view of the event’s usefulness/satisfaction and the basis for their judgment.
2. An evaluation of the whole iPP (not restricted to the timeline) regarding care goals, care plans, involvement in care decisions, experienced continuity of care and support for self-management.
3. An evaluation of the Patient Assessment of Chronic Illness Care (PACIC) questionnaire. We thought the PACIC, which builds on the Chronic Care Model, might be useful to capture patient experienced care quality. We therefore added a “Think aloud” session of the PACIC to explore its utility [45, 62]. The PACIC asks patients to estimate to what degree specific care characteristics, i.e., patient involvement, were present over the past six months of care. However, the informants explained that even if care was excellent most of the time, a single unfortunate incident at a critical moment could still have disastrous consequences, threatening the results of the entire chain of care. Furthermore, the PACIC did not differentiate between providers. E.g., care at provider X was always great, but provider Y, which they saw more rarely, was not. In both cases, the PACIC would capture this as excellent care most of the time, which seemed wrong. The first six respondents provided similar feedback. Hence, we omitted the PACIC in the subsequent interviews.

Interviews were conducted in the informant’s home, or in an office facility according to informants’ wishes by GB/DG (Connect study) in fall of 2011–2012 or GB/AH (TO-study) in 2012–2013. We interviewed the parents of the underage informant (parents views represent only one patient in quantitative descriptions). We transcribed interviews “ad verbatim.”

### Analyses

#### Evaluative assessment of the iPPs

Author pairs: 1) GB – AH, 2) GB - DG analyzed the interviews of the TO and Connect data respectively using a methodology recommended for framework development [59]. Each author pair first familiarized themselves with the material and then agreed upon a condensed synopsis with illustrative quotes capturing the salient themes. We mapped these to themes and sub-themes structured by the 4-stage PC-IC ideal.

#### Refinement and operationalization of the PC-IC framework

We developed refinements of the framework so that each “PC-IC stage” could be recognized more consistently across time, place and observers for each informant. Refinements were successively rephrased, merged/split and modified in light of the iPPs as we analyzed them. Refinements consisted of:

- PC-IC Ideal Descriptions: A short qualitative text of each of the four stages, which describes the desired “ideal” iPP attributes, aligned with literature underpinning each stage.
- PC-IC Key Questions: Formulation of open and closed questions designed to assist evaluation of the presence/ absence of desired attributes for each stage. We first formulated questions per case and successively rephrased to a more general form. E.g. Case: “The waiting-time guarantee of max eight weeks has expired, and my symptoms are worse. Why is my case still not prioritized?” = > General: “Did patients have to intervene to avoid or correct mistakes because planned/ expected care was not provided?”
- PC-IC Theoretical underpinning: A heuristic list of salient and relevant literature references linked to the ideals of each PC-IC stage.

We extracted the answers to each of the key questions in each PC-IC stage for each informant. We summarized the responses for all cases in a spreadsheet to ensure analytic consistency across informants.

## Results

### Goals

#### “What matters to you?”

Most life goals lie outside the scope of healthcare’s responsibility. However, when health issues are blocking the way forward towards a life goal, healthcare can be a vital enabler. The task at hand is to explore what the life goals may be, and then translate these into goals relevant for care.

We had already from the outset an understanding of the iPP as a goal-oriented process, and that we could not review PC-IC quality in the context of single visits or services. We nevertheless initially thought we would be considering sets of events defined by a single short-term problem, or a defining event identified as important. However, early in the interview series, we moved from discussing PC-IC in the context of discrete issues and events to discussing the entire iPP for the whole review period. The salience of the long-term goal was evident as informants repeatedly referred to evaluations of care relative to their overarching long-term goals.

One informant praises the care worker for honoring his long-term goal even when the child protection officer had taken legal steps to restrict the informant from seeing his son on account of his drug abuse.

*“(P) Person: I had to choose. Either I choose the booze or I choose my son. (…), it was really a simple choice. But of course, it was also a new experience when I met a person* [care worker] *who confronted me with this choice (…). It was at this point that I really woke up and saw the severity of my situation. That was when I started changing.” (…).*

*(I)Interviewer: How did you react to this* [the restrictions?] *(…)*

*P: “At first I was truly upset. But when a few more months had passed, I became really grateful. Because, if she had not made these demands, then I would not have made* [the necessary] *changes.”* Male, mental health and substance abuse issues.

The following is an example where a professional and a personal goal clashes in a decision process regarding the discontinuation of a medication the informant had been using for years. The change in treatment hindered the informant’s overarching goal: to be a good mother and to cope with her everyday life. The professional was concerned with adherence to professional treatment guidelines. The informant describes the impact on her life:

“*A restlessness, agitation. If I were to make a mind map, I would need 5-6 secretaries nonstop. So, sleeping quality is not good. It becomes exhausting in the end. In addition, you never feel quite awake. I passed 3 months or so without* [the medication]*. And then I went to see her and I told her that it was… I told her that I want the medication back. “Naahh” she said, we had to weigh effects against risks. Then I said: I do not care about risks. I will take that responsibility myself. It is my life. So, she said that if I assumed responsibility myself, it was ok…”* Woman, two mental health diagnoses.

However, these overarching goals were often not made explicit by the informant. Sometimes the informant did not verbalize “What matters” directly, but the important matter, i.e. the overarching goal, shines through in the dialogue.

Another informant repeatedly voiced a need to get his driving license back, but we understood why only at the end of the hour-long interview.

*“…That is why I now have asked for rehabilitation, but I didn’t get any. But, if I had gotten well enough to get my driving license back, I could have gotten quite a different “circulation” to my life.”*

*I: Where would you go if you got your license back?*

*P: “Well, I know this lady.* [From earlier, he describes her as someone who cares for him]*. She lives in the mountains. I could visit her there.”* Male, five long-term diagnoses.

In fact, when we asked informants directly what their goals of care were, some were surprised or puzzled, because goals of care were either too obvious (i.e., my goal is to survive my cancer), or the informant felt that health professionals should set health-related goals. However, shifting the question of goals into; “What matters to you?” gave a richer and more immediate insight into areas threatened by health issues [63, 64].

Two life areas: “To have gainful employment” and “Being supportive of others” were mentioned more often, and with more ardor. Rehabilitation and mental health services (4 persons) did formulate goals of care linked to life areas such as: “Being able to take care of my kids.” Other more biomedically oriented services did not mention life area challenges in the EHR, nor did patients expect this.

#### Biomedical goals

“What matters” was often, but not always a life-concern. It could also be a bio-medical issue, especially when the informant had experienced difficulties negotiating this as a legitimate issue with the care system. The informants did not focus much on other overarching biomedical goals which they felt were self-evident. Nine of the 19 informants had disagreed with healthcare professionals regarding the need for a diagnostic investigation, treatment or information at some point during the observation period. Informants reported that they needed to “stress the system” to be taken seriously.

*“If you don’t shout and scream then nothing will happen. (…) I have been incredibly lucky. All I can say is if I hadn’t been admitted to the “X-clinic” if I had gotten a stroke or a heart attack, I would have been dead now. I pestered them and elbowed myself into the hospital. I nagged and pleaded to be admitted. And the general practitioner, he admitted afterward that he didn’t think it* [my condition] *was as serious as it actually was.”* Male, coronary vascular disease.

These experiences caused informants great distress and feelings of helplessness. The informants described these as open disagreements, but providers reported only one of these cases in the EHR at the time of the disagreement. The informant’s version was, however, confirmed in the EHR later in six of nine instances.

#### Goals for self-management

Eight of the informants had received some support for self-management in their iPP. Nine expressed further needs for self-management support regarding their medication, support for physical exercise and training, diabetes follow up, stoma care, lymphedema care, benefits and support opportunities from social care including adapted employment, social skills training, and anxiety management. Lack of necessary arrangements and support for physical exercise/therapy, which is in the grey zone between self-management and treatment, evoked quite strong emotional responses in some participants. Again, mental health and rehabilitation services differ positively from the other providers in that they systematically document self-management support.

### Plans

#### The care plan

Informants understood the concept of a “personal care plan,” and 15 of 19 felt that this would have been useful, but they did not expect such a plan to be personalized (e.g., be adapted to their circumstances or priorities). Participants knew what was going to happen in the short term for single diseases such as cancer, cardiac arrhythmia and heart failure where a routine follow up plan was in place. Long-term planning of the iPP, including self-management support and proactive management of current or likely future health complaints, was not a focus for informants. Nor did professionals mention it in the EHR.

The informants judged the care delivery by the system’s loyalty to the plans and expectations they had been given. This informant based his expectations on the care planning process:

*“A great doctor admits me, (…). He says that there are so many issues to deal with here. We will look at your blood pressure first, then the “fibrillation.” Yeah – then we will look at your stomach, and then let all the rest wait until afterward. (…) Nahhh* [they] *didn’t look at my stomach. I thought it was a great plan he made. Dealt with one issue at a time,* [but] *then I was given some tablets, then I got better. (…) I was not done with all that either* [exercises]*, because I could have improved even more. That is when they sent me home.”* Male, five long-term diagnoses.

However, of the four persons with a written plan, three were unhappy. One informant described the plan as inflexible and feared to lose her right to treatment if she asked for personalization. The two others said that since the plan was not implemented, it was not useful.

*“I: Do you experience that there is coordination support for you?”*

*“P: I do not feel there is. I feel it is quite random. When the meeting arrives: Oh, now we are like “formal”, and now we are supposed to make “The Plan”. I get the notes from the meeting where it says what we are all going to do. However, what’s done is not always the same as what the note says. There is not really anyone who keeps tabs on anything. (…) If I don’t make it start, then things tend just to die out. And it is exhausting. I am actually the coordinator in all this. (…) That’s the point isn’t it, with seven persons in a team? It’s that they should give you feedback, and that they should be there in their domains and have completed this and that until the next time.”* Male, mental health and substance abuse issues.

#### Shared decision making

Involvement of patients, both in relation to the choice of interventions and tailoring of care to a personal context, was hardly mentioned in the EHR. Yet, five patients described consultations where they were actively involved in decision making, two in mental health services and three cancer patients. The other 14 informants did not experience this, nor did they expect it.

#### Interdisciplinary review

Several of the 14 participants with more than one long-term diagnosis recognized a need for multidisciplinary coordination. However, multidisciplinary review of interactions between different conditions occurred only once. This was upon specific request from an informant who realized that two condition-specific treatment plans conflicted.

Multidisciplinary coordination within one condition occurred for two informants. The informants took part in monthly (mental health) and bi-annual (parents to the child with multiple disabilities) planning meetings respectively. Even though the former of these two also had diabetes, they never discussed care for diabetes in the mental-health coordination meetings. One informant felt the team meetings were helpful, while the other characterized the team meetings as follows:

*“There are care planning meetings every six months. At these meetings, the participants typically “look at the floor” when tasks are distributed. The coordinator is very good, but it is clear that there are limitations to what she can do. At these meetings, we try to find out what should be done, who does what, and when, right? (…) But, it’s almost as if the participants want to crawl underneath the table, and not look at me. It is as P* [partner] *says, a parody. You’re so mad when you leave those meetings because there is no energy, no support. Quite to the contrary.”* Parents of a child with multiple disabilities.

### Care delivery

#### The system is responsible

All patients emphasized that they find care professionals, in general, to be caring, polite and compassionate. They often described good care as being treated as a person and not a diagnosis.

*“But for the most part, I have met, yes, persons you could call angels. (…) They* [the oncology nurses] *were so humane and warm and good. It was a good experience to come to them and feel their concern for you.”* Woman, breast cancer.

Despite the hard work and good intentions of the individual professional, slips occur, in which case informants perceived the “system” to be at fault. System shortcomings mentioned by participants were: stress, shortage of time, personnel or both, the traits of the organizational unit, or the system as a whole. Also, informants were “blind” to the organizational barriers and roles of different providers.

*“The worst about our care system, in which I include all psychiatric and substance abuse services and the whole package, it is when you are unable to voice your problems. When you are so far down there that you cannot make them listen, you are not seen, not heard. And I feel that it is in such a contradiction to what healthcare is really there to do. You feel inferior, you feel invisible, and you feel so lost. It is as if you’re not worth anything.”* Male, mental health and substance abuse issues.

This focus supported our growing awareness of the entire continuum of care, i.e., “the system” as the agent delivering care instead of individual professionals. The patients reviewed their iPP regarding how the system creates and supports a “common” understanding of what the goal for this person’s iPP is, what the care plan is aiming to achieve, and what each contributor’s role/task relative to that goal is. It is no longer a question of each professional doing the right thing within his/her professional domain. It is more a question of whether the professional actions align with the other professionals, the care plan, and the overarching goal.

#### Delivered according to plan

The expectation of care delivery according to plan was strong with all informants. This was also the issue that most demonstrated the difference between event-based and process-based evaluation of care. The informants based their expectations on what their providers had told them would happen. If the next provider in the chain of care did not comply with the plan, this was cause for a range of reactions, from mild acceptance of the care systems fallibility to strong emotional responses towards a flawed and unreliable system.

*“I used nine days to get the prescriptions I should have been given so that I could start treatment* [anti hormonal adjunct treatment for breast cancer] *in January. And it was a little bit… In the end I had to say: “Who is responsible here?*” *At that point I had gone to the mammography center, the oncology department, and the oncology outpatient clinic… And in the end, I said to them – I am NOT leaving. Now you MUST find me a doctor who can listen to my challenge which is to get the right prescriptions for the medications that I should have begun taking yesterday.”* Woman, breast cancer.

Eleven of 19 informants reported failures of delivery of planned care in their iPP. The most common were missing invitations and referrals to planned clinical assessments and examinations, missing prescriptions and miscommunications. One informant, who had been assigned a professional coordinator, described how the implementation of planned activities rarely proceeded as expected unless they “pushed for action.”

#### Informational continuity

All informants could confirm that they had to tell their story over and over. In some cases, relevant information was not available in a timely manner. For example, the hospital discharge summary for one informant arrived a month after discharge, long after both the nursing home and the GP had adjusted medications many times. Informants did not expect to meet the same healthcare professionals every time. Informants acknowledged, rather good-naturedly, that professionals vary regarding their assessments of the clinical diagnosis and care. This causes confusion but is perhaps also inevitable.

*“There are as many opinions as there are doctors. The doctors are of course wonderful, but it was truly interesting to come straight from the doctor’s consultation to the oncology nurses who said: Don’t listen to the doctors. We are the ones who know!”* Woman, breast cancer.

### Evaluation

There was no evidence in the EHR that suggested that patients and/or professionals had evaluated goal attainment in any way. Informants confirmed, that they had not participated in any assessment of goal attainment, or care evaluation in general. Such an evaluation would ideally be directed at the care coordinator/case-manager or to the care team, to support the adjustment of the care plan before the next care cycle.

### From ideal to framework – What does our study add?

We incorporated the findings into our evaluation framework of the PC-IC process through the operationalization table. The following ten quality attributes are additions to the PC-IC framework:

1. The unit of evaluation is the long-term iPP process, not the care-event, or series of care events.
2. The iPP process consists of four stages, each with distinct desired quality features.
3. All stages build on each other, starting with the overarching goal based on the person’s answer to the question “What matters to you?”
4. Identifying “What matters to you?” may not be straightforward. Building trust and being creative in inferring goals from other statements may be necessary. What matters may vary widely from a biomedical problem to a life area.
5. Not all life-goals are health-care concerns. Therefore, the overarching goal must be translated, in an open and non-judgmental process, into realistic and relevant goals of care. This type of negotiation requires good communication and balancing skills.
6. The range of skills and capabilities that need to be involved in the iPP, flow from the goals relevant for care. These goals lead to the identification and involvement of necessary skills and competencies wherever they can be found.
7. The care planning starts with assessing and negotiating the overarching individual goals, and proceeds to build on relevant evidence-based guidelines, not the other way around.
8. Care integration is achieved when all the different skills and competencies are effectively orchestrated into supporting the goals of care negotiated between the patient and the healthcare provider.
9. The quality of the care plan depends on how well it supports the overarching goal. The quality of care delivery depends on how well it provides the expected and planned care.
10. Goal oriented care must include a goal-evaluation. If providers do not assess success or failure, then no learning or adjustment will occur (Table 2).

**Table 2:** Characterization of the four stages of the Person-Centered Integrated Care (PC-IC) cyclical process for evaluation of individual Patient Pathways (iPP)

Description of ideal care	Key questions	Supporting literature
1. Goals		
The unit of observation is the long-term iPP. The ideal iPP should meet the overarching personalized goals, which reflect “What matters to the person.” The overarching goal defines the scope of the care plan. It includes; • an empathic and sensitive effort to understand what the person’s needs, values and preferences are • Negotiating and documenting goals of care that are relevant, realistic and observable. • eliciting and recording the person’s resources as a partner in decision making regarding health and wellbeing Overarching personal goals can be broken down into supporting sub-goals in a goal hierarchy. In case of conflict between professional recommendations and personal goals, the person’s goals should prevail, unless they compromise legal or ethical principles. In case of legal or ethical barriers, a documentation of how the conflict was explored with the person and what conclusions were reached is desirable.	How do persons express “What matters to them?” What are the patients’ perceptions of healthcare’s reaction to his/her articulation of “What matters to them?” Did the informants express unmet needs, values or preferences? If there were unmet needs, conflicting view of goals, were these described or explained in the EHR? What needs for self-management support do informants voice, and were these needs met?	• Goal-oriented care [31, 35, 83] • The informed, active patient [84] • Patient-centered care [33] • Person-Centered care [85] • People centered care [86] • What matters to you? [63, 64] • Self-management support, patient involvement, and engagement [87] • Self-determination theory [88] • The ethics of authenticity [89]
2. The care plan		
The care plan is based on a multidisciplinary review of the goals from step 1. The first step is to identify skills and competencies needed to achieve these goals. There are no organizational limits regarding whom to include in the iPP plan. The decision process should involve all relevant providers and the patient/caregivers as far as possible to promote engagement, realism, and ownership of the plan. Plans take into account and document the patient’s resources as a partner in the collaborative work for health and wellbeing The care plan should ideally: • Be committed to and aligned with personal goals • Be evidence-based • Include a multidisciplinary review in cases of multimorbidity • Ignore organizational boundaries • Describe self-management and its support • Describe monitoring for exacerbations • Include a crisis management plan • Include a time and method for goal evaluation. • Include community resources that can be leveraged to help meet goals	Was a written or verbal care plan described in the EHR, or by the patient? What are the patient’s descriptions of involvement and engagement in care planning and shared decision-making (SDM)? What are the EHR descriptions of SDM? Do care plans include the following components: • Reference to personalized goals? • Self-management support? • Multidisciplinary review whenever relevant? • Monitoring for exacerbations? • Emergency or crisis management? • Checkpoints for evaluation of goal attainment, or goal revision?	• Shared decision making [90, 91] • Prepared proactive healthcare team [84] • A personalized care plan [50, 92] • Decision support [84] • Evidence-based medicine [91] • Self-management support, patient involvement, and engagement [87]
3. Care delivery		
Care delivery builds on the care plan from step 2. The delivery of care is a system property, not a feature of individual professionals. The care system should identify the resources necessary to reach overarching goals irrespective of organizational boundaries and responsibilities. A marker of high quality care delivery is that the person feels that he/she is seen, heard and recognized as a person. Seamless care delivery depends on the recruitment of the resources that will implement the care plan with attention to Continuity of Care, and it’s organizational, informational and relational dimensions as described by Haggerty [93]. Haggerty’s “relational continuity,” serves primarily to elicit and communicate “what matters” to the system. Thus, we argue that “relational continuity” is a kind of informational continuity.	• Was the care plan operationalized to show where, when and who would provide their care? • If so: What was the perceived usefulness of such operationalized plans? • Did patients experience unexpected care events? • Did patients have to intervene to correct mistakes because expected care delivery was not provided? • Were patients directed to community resources outside of the healthcare system such as peer support organizations or social services? • What were patient’s statements regarding the organizational, informational and relational continuity of care across their iPP?	• Delivery system design [84] • Community resources [84] • Care pathways [94] • Continuity of care [93]
4. Goal attainment		
The iPP success is measured by the degree of goal attainment of goals set in step 1. Goal evaluation enables learning and adjustment of the iPP for the next turn of the care cycle.	• Did they plan and assess goal attainment? • If so: Did they adjust the future care plan according to lessons learned?	• Health and Functional outcomes [84] • Goal oriented care [31, 35, 83, 75]

Descriptions of ideal care, key questions, and literature underpinnings to support a consistent evaluation of care across observers and informants

### Summary of findings

Our contribution is the description of a goal-oriented PC-IC cyclical process framework for evaluation purposes.

An empathic and sensitive exploration of “What matters” is the basis for understanding what the “overarching goal” for the iPP is. From there flows a set of negotiated goals relevant for care, the care plan, care delivery, and care evaluation. We found that the informants assessed their care in terms of their long-term life goals, although some also focused on biomedical goals. Care planning was common for short-term single diseases, but not for long-term multimorbidity. Informants viewed the “care system”, not the individual professional, as responsible for care delivery. The application of the PC-IC process framework to patient experiences showed that providers do not record nor share goals, care plans, monitoring of care delivery nor goal evaluation for persons with multimorbidity across the care system.

We were able to demonstrate the fragmented and profession-centric nature of current care delivery. Based on the lessons learned, we characterized each of the four PC-IC stages using: 1) ideal descriptions, 2) key questions and 3) supporting literature references. The resulting framework allowed us to evaluate the PC-IC aspects of 19 multimorbidity iPPs consistently.

## Discussion

### The iPP quality assessments

As PC-IC is high on the political agenda, the need to support change management towards PC-IC is substantial. The “PC-IC process model” that was proposed and refined in this study was intuitive to informants. With this mental model, persons were able to express what mattered most to them, and assess care delivery relative to their personal goals and care plans (or lack thereof). We identified issues that other studies do not commonly identify as challenges in the literature on PC-IC:

1. **The salience of biomedical goals.** Informants were most concerned and upset with immediate unmet medical needs and slips in expected care delivery. It was surprising to us that this was a substantial finding. In most of the person-centered care literature, there is a focus on the patients’ needs as individuals [5, 65]. Our findings highlight the importance of ensuring that the person’s biomedical concerns are not lost in the exploration of “What matters to you?” [33, 66]. The numerous patient safety complaints in this study seemed both legitimate and important. With the internet revolution, patients are both more informed and connected to relevant resources that can support their evaluation of biomedical issues [67]. Other studies also suggest that persons with multimorbidity experience more quality challenges than persons with only a single condition [3, 68, 69]. Cook [6] claims that all healthcare is by nature fragmented and that bridges are constantly “invented” to ensure that the right thing happens at the right place and time. He claims that gaps occur when “…*conditions overwhelm or nullify the mechanisms practitioners normally use to detect and bridge gaps*” [6]. It may be that the complex needs of our informants routinely “overwhelm” normal care practices. Although patients cannot be expected to assess all aspects of technical quality, it is likely that the introduction of a regular goal-oriented PC-IC process evaluation, could detect obvious slips in the technical quality of care.
2. **The overarching goal defines patient-experienced quality.** Another significant finding is the lack of attention to the ensemble of the person’s needs and challenges and the failure to share overarching goals and care plans with all relevant parties. We found the ideal PC-IC plan to be defined by 1) the person’s long-term goal and 2) to include a system-wide perspective, “blind” to organizational arrangements between providers. While it is encouraging to note that mental health and rehabilitation services comply more often with PC-IC ideals, even these services often limited their scope of the care plan to one main condition. Our findings are in line with other reports which describe care systems as focused on professional and diagnostic centric goals [4, 48, 70–72]. Health professionals focus on disease control, while patients link goals to meaning and well-being (e.g., employment, family) [22, 65, 72, 73].
3. **Goal-oriented process, not event evaluation**. Our findings differ from those derived from tools and frameworks for PC-IC that typically focus on care event(s), or events by a given provider [1, 40–45]. The essence of person-centeredness is to allow the answer to the question: “What matters to you?” to define the quality of the whole care process. We acknowledge that a need for a more focused and time-limited evaluation can be legitimate. A review of limited events, with their concomitant sub goals and sub-plans can accommodate this. Yet, we would argue that it is essential that event sub-goals remain properly aligned with the overarching goals of the person and are ranked within the entire set of sub-goals and activities going on.
4. **The translation** of the overarching goal into relevant and realistic goals of care is a complex negotiation and balancing act. In this material, the negotiation process regarding the legitimacy of biomedical goals was frustrating for the informants. There were also examples of conflicts between the explicit person and professional goals. The most glaring finding is, however, the missing negotiations, indicating that the actionable goals are taken to be self-evident and not in need of negotiation.

Our results indicate that the PC-IC process framework for the evaluation of care has the capability of capturing; 1) the quality of a goal identification process, and 2) the system’s (in)ability to share goals, care plans, delivery monitoring and goal evaluation across relevant contributors. The PC-IC process framework can thus provide a basis for the development of new qualitative and quantitative tools, which can support management of change in the direction of PC-IC ideals.

### Development of PC-IC evaluation tools

Measurement is one key to system change: It gives the basis for assessment of where we are at, and can represent a guide to adjustment and improvement efforts [74]. The process of capturing patient stories, as we did, is too cumbersome to be pragmatic in large-scale health service evaluations. However, the informants´ intuitive grasp of the framework is encouraging and attests to the feasibility of pursuing this line of inquiry.

This methodology may form the basis for the development of evaluation tools built on both qualitative and quantitative methods. To be useful, the process needs to be simplified, both regarding administration and analysis. We see two apparent routes of further development: 1) Interview guides for personnel who are familiar with the person’s history, based on the key questions. 2) Structured questionnaires that gather qualitative or quantitative responses that rely on the person’s recollection of his/her history. Such surveys can, with enough explanation, be filled directly by the person with the support of health personnel if necessary. Qualitative statements may provide useful feedback directly to care providers. For aggregate data at the population level, data must be quantifiable. Quantitative measurement instruments of person-specific goals [75–77] can be merged with our four-stage PC-IC care process to create such tools.

### Implications for practice and the EHR

By endorsing the PC-IC process ideal as an evaluation tool, we are also making a normative statement about care for patients with multimorbidity. A growing knowledge base supports the importance of PC-IC coupled with a goal-oriented process [14–21]. However, even though the field is learning rapidly, the literature in this area is still widely heterogeneous and inconclusive concerning which care components are necessary and sufficient, for whom and in what contexts [19, 78, 79]. Our recommendations therefore still need further validation regarding implementation and effect on iPP experiences, health outcomes, and cost-benefit results.

An essential tool to support cohesiveness across the care system is the EHR. In our Norwegian context, EHRs are available in all GP, nursing services, and secondary care settings. However, the EHRs mirror strict organizational, legal and economic silo patterns. Except for limited two-way electronic messages and communication such as the exchange of discharge/referral letters, there is no standard electronic interface which supports the interactive and updated sharing of goals, care plans, care delivery monitoring or goal evaluations across organizations. It seems logical, although data are still sparse on this issue, that such electronic tools would be helpful [80, 81].

### Strengths and limitations

The combination of EHR derived summaries and interviews was an effective way of gaining insights into complex care stories. Even with a considerable variation in individual conditions, we experienced a sense of saturation for all four areas of assessment. The findings resonate with both theory and other empirical studies in the field, lending credibility, and relevance to the study.

This study examines one domain of quality of care. Quality is a multi-dimensional construct, and there are many other quality domains not evaluated here [82]. Notably, patients cannot be expected to assess the area of technical quality. Our method of evaluating quality should not exclude the review and assessment of other quality domains in the iPP.

Due to resource limitations, we made iPP timelines and summaries based on the GP, hospital, and nursing service health records only. Ideally, we should have included all health services and all service providers, as well as informal caregivers. It would also have strengthened the study if we could have interviewed the child with a disability in addition to his parents.

This study raises concerns regarding the quality of care for persons with multimorbidity, but the small sample size does not allow for generalizations. There is also a need for validation of our framework in other settings and conditions. Persons with only a single condition or less complex care needs may not recognize these concerns as readily.

## Conclusions

This paper provides insights and methodology that may support quality evaluation towards a goal and process-oriented PC-IC ideal of chronic care. Use of this evaluation method revealed important weaknesses, commonly associated with fragmented and discontinuous care for multimorbid informants. This paper highlights how gaps in care that are invisible with an event-focus on quality of care, becomes visible when we use a long-term goal-oriented PC-IC process as an ideal.

## Additional file

Additional file 1: Interview guide. (DOCX 15 kb)

## Abbreviations

EHR: Electronic Health Record
GP: General Practitioner
iPP: Individual Patient Pathway
Multimorbidity: Multiple long-term conditions and/or complex healthcare needs
PC-IC: Person-Centered Integrated Care
TO: Troms-Ofoten (a region in Northern Norway)

## Footnotes

1 Connect study only

2 TO study
