# Supplementary material for: A person-centered integrated care quality framework, based on a qualitative study of patients’ evaluation of care in light of chronic care ideals
Source: BMC Health Serv Res. 2018 Jun 20;18:479. doi: 10.1186/s12913-018-3246-z (PMC6011266; doi:10.1186/s12913-018-3246-z)
Supplement: Supplementary file 1 — Interview guide. (DOCX 15 kb) [file 12913_2018_3246_MOESM1_ESM.docx]

# Interview guide, English translation:

(For original Norwegian version, please contact corresponding author).

## English translation - Interview Guide - The individual Patient Pathway

The interviewer reviews the patient's medical history, as presented in the synopsis based on the electronic health records, together with the patient at the start of the interview.

### An overview of your health service visits during this period

Are there any important healthcare events that are missing?

Is any of the information I have shown you wrong?

Do you want to add any visits to services outside the conventional health service that was important to you at in this period? Examples: dental services, physiotherapy, alternative therapy, etc.

Think of your overall care. How did you experience the collaboration with the health services during this period?

### Key health service events in the review period

What events were important to you? Describe why they were important.

What events were particularly satisfactory - describe these and why they were satisfactory.

What events were particularly challenging - describe these and why they were challenging.

Did these events have consequences for you?

### Goals and plan for treatment, and follow up of treatment over time

For long-term illness or conditions, it matters that both caregivers and patients have a common view on what the goals of care are and how care will be delivered.

What do you understand by the term “goals of care”?

The “goals of care” will depend on how your condition(s) effect(s) your life, what is realistic to achieve and what matters most to you in your life.

Are you able to formulate what you consider your goals of care the last year - can you explain why?

Have you and your providers discussed goals for your current/future care?

### Choice of treatment

Have your providers told you or discussed with you whether there are different treatment options for your condition(s) that you may choose among?

To what extent do you feel that health professionals know your personal preferences when they choose or recommend a treatment?

To what extent do you feel health workers actively include your significant others in discussion/information when they choose or recommend a treatment?

In treatment of acute illness, health professionals often have to make quick decisions on your behalf. However, in long-term illnesses/conditions, it is useful to schedule follow up over time. Here we are referring to scheduling multiple contacts over time and perhaps also coordination across different parts of the health service that are responsible for different parts of the follow-up.

How do you understand the term follow-up?

Do you know if a follow-up plan has been made for you?

What do you think might be the potential benefits from a follow-up plan?

To what extent do you feel health professionals discuss with you and take into account your personal situation when they make a follow-up plan for you?

To what extent do you feel health professionals involve your significant others when they make a follow-up plan for you?

If there is no follow up plan, do you feel that health workers actively contact or summon you to discuss how things are progressing after a treatment has been recently started or ended?

### Integration between services and transition between services

You've probably experienced being referred from one part of healthcare to another for assessment or treatment. Now we wish to focus on transitions between services in healthcare (e.g., from GP to hospital or from Department A to Department B). We want to look at the coherence of services, both concerning how they are organized, how information travels between the services and how your needs have been taken into account.

(Organizational continuity)

When you were referred to another unit in the health service, how was the decision made and how was it communicated to you?

Did the referral proceed as you had expected/or were explained?

If a referral did not proceed as it should, did this have any consequences for you?

(Informational Continuity)

Did you get the sense that the providers that you have met had the necessary knowledge of your medical history (previous test results, past decisions, etc.)?

If you experienced the provider was not entirely up to date on your history, did this have any consequences for you?

(Relational continuity)

Did you get the sense that the providers that you have met, from your point of view, have the necessary knowledge about you and your situation, to understand your ability to be actively involved in your treatment and to understand what was important to you in this situation?

Do you feel that health professionals ask you how the ongoing treatment fits your life situation and what matters to you?

If you experienced a lack of knowledge about you - did this have any consequences for you?

### Self-care for your conditions

For most long-term conditions, there are some things you can do yourself to make it easier to live with the disease and to prevent complications or recurrence of the disease.

Do you feel that health professionals informed you about your self-management opportunities for well-being and prevention of exacerbations?

Have you been offered or received information/training to develop your self-management skills? For example; courses, information materials, referral to peer support groups, etc.

Did you receive too much/too little information/education? Was information provided when you needed it in a timely manner?

Do you feel that health professionals discuss with your significant others their opportunities to contribute so that you are all able to cope as well as possible with your condition?

### Self-care - general health and wellness

Life with long-term conditions can present challenges in complying with general health and lifestyle advice.

Have you been offered or received information/education about what you can do to safeguard your general health in your current situation? For example, lifestyle, diet, physical activity, etc.

Do you feel that health professionals discuss with your significant others what you can do together to safeguard your general health in the current situation?

### The vision for a course of treatment?

If you think back on your patient journey - and allow yourself some "hindsight" - how would you describe the ideal healthcare pathway for you? Please provide the reasoning behind your answer if you feel you can.

Finally - do you have any comments/questions to us who are doing this study?

Do you have anything you want us to convey to the hospital management?

Thank you for your help and best wishes to you!
